# Crop residues exacerbate the negative effects of extreme flooding on soil quality

**DOI:** 10.1007/s00374-017-1214-0

**Published:** 2017-06-19

**Authors:** Antonio R. Sánchez-Rodríguez, Paul W. Hill, David R. Chadwick, Davey L. Jones

**Affiliations:** School of Environment, Natural Resources and Geography, Bangor University, Bangor, Gwynedd LL57 2UW UK

**Keywords:** Iron, Nitrogen mineralization, Nutrient cycling, Phosphorus, Methane, Nitrous oxide, Waterlogging

## Abstract

Extreme flood events are predicted to have a negative impact on soil quality. Currently, there is a lack of information about the effect of agricultural practices on soil functioning and microbial processes under these events. We hypothesized that the impact of flooding on soil quality will be exacerbated when crop residues are present in the soil as they will induce more extreme anaerobicity. A spring extreme flood event (10 °C, 9 weeks) was simulated in mesocosms containing an arable sandy-loam soil low in nutrients. The main treatments were (1) with and without flooding and (2) with and without maize residue addition (8 Mg ha^−1^). We monitored changes in soil chemical quality indicators (e.g. pH, salinity, Fe^3+^, P, C, NH_4_^+^, NO_3_^−^ and organic N), greenhouse gas (GHG) emissions (CO_2_, CH_4_, N_2_O) and soil microbial community composition (PLFAs) during a prolonged flood period (9 weeks) and an 8-week “recovery” period after flooding. In comparison to the other treatments, flooding in the presence of crop residues resulted in a dramatic drop in soil redox potential. This was associated with the enhanced release of Fe and C into solution and an increase in CH_4_ emissions. In contrast, maize residues reduced potential nitrate losses and N_2_O emissions, possibly due to complete denitrification and microbial N immobilization. Both flooding and maize residues stimulated microbial growth and promoted a shift in microbial community composition. Following floodwater removal, most of the soil quality indicators returned to the levels of the control treatment within 5 weeks. After this short recovery phase, no major impact of flooding could be observed on plant growth (maize pot-grown). Overall, we conclude that both extreme flooding and management regime negatively impact upon a range of soil quality indicators (e.g. redox, GHG emissions); however, the soil showed high resilience and recovered quickly after floodwater removal. Further work is required to investigate the impact of repeated extreme flood events on soil quality and function over longer timescales.

**Electronic supplementary material:**

The online version of this article (doi:10.1007/s00374-017-1214-0) contains supplementary material, which is available to authorized users.

## Introduction

Although controversial, there is increasing evidence to suggest that climate change is already causing an increased frequency of extreme flood events in some regions of the world (Pall et al. 2011; Slater and Villarini 2016). However, as warming temperatures amplify the atmosphere’s water holding capacity, it is widely agreed that the occurrence of extreme precipitation and flooding events will increase (WMO 2013; Volosciuk et al. 2016; Yuan et al. 2017). This is likely to have a major impact on food security, ecosystem functioning and human health (Mirza 2011; Tong et al. 2016). It is therefore important to understand how these extreme events may impact upon soil functioning and how we may be able to better manage soils to prevent a loss in ecosystem service provision.

While many soils on floodplains regularly suffer from inundation, the increased magnitude of flood events means that new areas with no recent history of flooding are now becoming severely affected (Yellen et al. 2014). In addition, these extreme floods can result in floodwater remaining on the land for several weeks or months, and in some cases up to several metres in depth (Natural England 2014). In terms of a loss in natural capital, the worst-case scenario occurs when the floodwater is moving laterally across the landscape facilitating the erosive loss of large quantities of topsoil (Li and Wei 2014; Boardman and Vandaele 2016). Typically, however, floodwater remains stationary on the soil, although this can still be subject to wind-driven turbulence within the overlying water column. Surface flooding can be expected to cause considerable losses in soil quality and plant productivity as well as inducing changes in nutrient cycling, meso- and macro-faunal abundance and microbial community composition (Bossio and Scow 1998; Niu et al. 2014). It is also likely that the impact of flooding on soil quality will be heavily influenced by both past and present management regimes (e.g. fertilizer additions, presence of organic residues, plant cover). To minimize the consequences, work is therefore needed to identify management practices which may exacerbate the negative effects of long-term flooding, and identify strategies that may increase resilience.

Sustainable agricultural practices typically involve the use of crop residues as a soil amendment to increase organic matter content, promote soil C sequestration, provide erosion control and improve nutrient cycling. Crop residues are rich in C but also represent a source of other nutrients (e.g. N, P). The availability of these nutrients to plants and microorganisms, however, depends on their relative concentrations and speed of turnover. In most arable systems, organic residues in the form of straw or stubble are largely incorporated into the soil at the end of the growing season or in the spring. These residues typically contain low levels of N and P which may ultimately lead to net immobilization and a reduction of available nutrients in the soil (Damon et al. 2014). In some situations, this may have positive benefits as it may reduce NO_3_^−^ leaching; however, this effect is not always observed (Doring et al. 2005; Hansen et al. 2015). The effect of cereal residues on greenhouse gas emissions (GHG), particularly N_2_O fluxes, is also controversial (Chen et al. 2013). Specifically, they may increase nitrification under aerobic conditions (Frimpong and Baggs 2010), and provide energy for denitrifiers facilitating denitrification under anaerobic conditions, both of which can produce N_2_O (Davidson 1991). Crop residue quality appears to be inversely related to N_2_O emissions with high C:N ratio residues producing lower emissions (Li et al. 2013). In addition, CH_4_ emissions may also result from C-rich crop residues, particularly under waterlogged conditions (Devevre and Horwath 2000).

In the context of current climate change, the main aim of this experiment was to investigate how soils with no previous history of flooding respond and recover from an extreme flood event. In addition, the secondary aim was to investigate whether the presence of crop residues accelerated the decline in soil quality. We hypothesize that high C-to-N ratio cereal residues would reduce N_2_O emissions but increase CH_4_ emissions during flooding. Further, we hypothesized that shifts in soil chemistry and microbial community composition during flooding would cause lasting damage to the soil leading to a loss of function in terms of its ability to support primary productivity.

## Materials and methods

### Soil and maize residue characterization

A sandy textured Eutric Cambisol (FAO 2015) was collected from the surface (0–20 cm depth) Ahp horizon of an arable field located in Worcestershire, England (52° 6′ 23.5 N, 2° 13′ 39.5 W; Fig. S1). The soil was typical of those inundated during the extreme flood event experienced in the winter of 2013–2014 (Fig. S2 A; Met Office 2014; Muchan et al. 2015), but was collected above the flood line and had no recent history of flooding (Fig. S2 B).

To characterize the soil, replicate batches of soil (*n* = 3) were air-dried (25 °C) and passed through a 2-mm sieve. The pH and the electrical conductivity (EC) were determined in a 1:2.5 (*w*/*v*) soil:distilled water suspension with standard electrodes. The pH was 6.4 ± 0.1 and the EC was 1.4 ± 0.1 dS m^−1^. Soil solution was extracted from the soil using the centrifugation-drainage method of Giesler and Lundström (1993) and soluble N determined spectrophotometrically on a PowerWave-XS microplate reader (BioTek Instruments Inc., Winooski, VT) according to Mulvaney (1996) for NH_4_^+^ and Miranda et al. (2001) for NO_3_^−^. The NH_4_^+^ and NO_3_^−^ concentrations were 1.0 ± 0.2 mg N l^−1^ and nitrate 63.4 ± 4.4 mg l^−1^ respectively. Total C and N were determined using a TruSpec® analyser (Leco Corp., St Joseph, MI) and found to be 3.03 ± 0.01 g C kg^−1^ and 0.67 ± 0.01 g N kg^−1^ (C:N ratio, 4.5). The maize residues (stubble) were collected at the end of the growing season after crop senescence (Fig. S3). The residues were air-dried (20 °C) and cut up into 1–2-cm-long pieces for use in the experiments. The total C content of the stubble was 495 ± 1 g C kg^−1^ and the total N was 5.22 ± 0.08 g N kg^−1^ (on a dry weight basis; C:N ratio, 94:1).

### Experimental design

Sixteen transparent polypropylene containers (11 × 8 cm base, 27 cm high; Lock & Lock Ltd., Seoul, Republic of Korea) without drainage holes were filled with 900 g of field-moist soil sieved to pass 1 cm. Chopped pieces of maize residue were then mixed with the soil in half the mesocosms at a rate of 8 Mg ha^−1^ (7 g mesocosm^−1^), reflecting typical farm practice (AHDB 2015). For the non-destructive removal of soil water, a Rhizon® sampler (Rhizosphere Research Products, Wageningen, The Netherlands) was inserted into the centre of the soil in each mesocosm at an angle of 45° and depth of 7 cm. The soil was left unplanted to simulate the spring fallow period of the maize agronomic cycle (AHDB 2015). The containers were transferred to a constant-temperature room and incubated at 10 °C in the dark to simulate winter/spring conditions. Dark conditions were used to simulate the restricted light conditions experienced in spring floods (due to particles in the water column; Fig. S2). After a week of pre-flood incubation, distilled water was added to 8 of the 16 mesocosms, 4 with maize residue and 4 without maize residue, to give a 10-cm floodwater depth above the soil surface. The experiment therefore had 4 treatments in a factorial design:

1. Control or no-residue addition without flood (NR)
2. No-residue addition with flood (NR + F)
3. Maize residue addition without flood (MR)
4. Maize residue addition with flood (MR + F)

There were four replicates per treatment. The floodwater was maintained at a constant depth (by addition of distilled water) for 9 weeks after which it was removed. Soil moisture in the non-flooded treatments was maintained at 70–80% of field capacity by adding distilled water weekly (determined via weight loss).

To simulate a late spring flood recovery phase, all the mesocosms were transferred to a climate-controlled Fitotron® plant growth cabinet (Weiss Technik UK Ltd., Ebbw Vale, UK) maintained at 15 °C in the day and 10 °C at night, with a photoperiod of 18 h day^−1^, light intensity of 350 μmol m^−2^ s^−1^ and relative humidity of 70%, for 8 weeks. The previously flooded mesocosms were allowed to naturally dry out to the level of the unflooded treatments. Watering was not applied to all treatments during the last 2 weeks of the soil recovery stage to reduce the soil moisture prior to undertaking the final crop establishment phase of the experiment.

To evaluate the post-effect of flooding on plant performance, soil from each flood mesocosm was recovered and placed in individual 1-l plastic pots. Four pre-germinated (72 h) maize seedlings (*Zea mays* L.) were then planted in each of the 16 pots. After 7 days, the plants were thinned to leave two plants per pot. To fertilize the plants, a nutrient solution containing 10 mM KNO_3_ and 15 mM KH_2_PO_4_ was added to each pot at a rate of 40 ml pot^−1^ week^−1^ (5.6 kg N ha^−1^ and 19.6 kg P ha^−1^, in total). The plants were grown for 4 weeks at which point they were harvested. The conditions in which the maize plants grew were the same as those detailed for the late spring flood recovery phase except the temperature during the day (20 °C).

### Soil chemical indicators

Soil solution using a Rhizon® sampler (0.15 μm pore size) and floodwater using a pipette were collected on a weekly basis during the flood experiment. pH was measured using a Model 209 pH meter (Hanna Instruments Ltd., Leighton Buzzard, UK) and EC using a Jenway 4520 conductivity meter (Cole-Parmer Ltd., Stone, UK). Fe (Loeppert and Inskeep 1996), P (Murphy and Riley 1962), NH_4_^+^ (Mulvaney 1996) and NO_3_^−^ (Miranda et al. 2001) were measured by spectrophotometry on a PowerWave-XS microplate reader, and total dissolved organic C (DOC) and total dissolved N (TDN) using a Multi N/C 2100/2100 analyser (AnalytikJena AG, Jena, Germany). Dissolved organic N (DON) was calculated by subtraction of NO_3_^−^ and NH_4_^+^ from the TDN value. The last sampling was performed 13 weeks after flooding. Oxidation-reduction potential at a soil depth of 3 cm was determined periodically throughout the experiment using a WTW SenTix® probe (Xylem Analytics Ltd., Letchworth, UK). The potential losses of nutrients were calculated as the amount of nutrient released into the soil solution and floodwater (*C*_release_) as follows:

1 $$ {C}_{\mathrm{release}}\left(\mathrm{mg}\;{\mathrm{container}}^{-1}\right)=\left[{C}_{\mathrm{sol}}\times {V}_{\mathrm{soil}}\times \varTheta \right]+\left[{C}_{\mathrm{flood}}\times {V}_{\mathrm{flood}}\right] $$

where *C*_sol_ is the concentration of nutrient in the soil solution, *C*_flood_ is the concentration in floodwater, *V*_soil_ and *V*_flood_ are the volume of soil (0.70 l) and floodwater (0.88 l), respectively, and *Θ* is the volumetric water content (0.5 cm^3^ cm^−3^).

### Soil greenhouse gas emissions

Greenhouse gas (GHG) samples were taken weekly during the flood experiment except for the 10th week after flooding in which two samples were taken (before and after removal of the water). At each sampling event, gas-tight lids containing a Suba-Seal® gas sampling port (Sigma-Aldrich Ltd., Poole, UK) were placed on the mesocosms. Headspace gas samples from each mesocosm were then removed using a syringe and placed into pre-evacuated vials at 0 and 1 h. Methane, CO_2_ and N_2_O concentrations in the vials were determined by gas chromatography using a Clarus 500 GC with a Turbomatrix (HS-40) autoanalyser (PerkinElmer Inc., Waltham, MA). CH_4_ and CO_2_ were measured with a flame ionization detector (FID) connected to a methanizer and N_2_O by a ^63^Ni electron-capture detector. Daily fluxes were estimated as the slope of the linear regression between concentrations at the two times taking into account the temperature and the ratio between chamber volume and soil surface area (MacKenzie et al. 1998). Cumulative fluxes were estimated by linear interpolations between two measurements of daily fluxes and adding the amount to the previous cumulative total. The global warming potential (GWP) of CH_4_, CO_2_ and N_2_O was estimated in CO_2_ equivalents by multiplying the cumulative fluxes at the end of the experiment by 34-CH_4_, 1-CO_2_ and 298-N_2_O before summing them, as a means of comparing the GWP of the different gases, and to be able to explore the effect of the different treatments on the total GHG emission (IPCC 2013).

### Soil microbial community analysis

After the flood stage and at the end of the experiment, soil samples (25 g) were removed from each mesocosm and stored at −80 °C for microbial community analysis. Phospholipid fatty acid analysis (PLFA) was undertaken according to the method of Bartelt-Ryser et al. (2005) with taxonomic groups ascribed to individual PLFAs using the Sherlock® PLFA Method and Tools Package (PLFAD1; Microbial ID Inc., Newark, DE). A total of 70 fatty acids were found in our soil samples but we only chose those that represented more than 0.5% of the total PLFA for biomarker and taxonomic group annotation (Ratledge and Wilkinson 1988; Kieft et al. 1994; Paul and Clark 1996; Olsson et al. 1999; Zelles 1999; Madan et al. 2002; Niklaus et al. 2003; Bartelt-Ryser et al. 2005; Table 1).

**Table 1:** Fatty acids (>0.5% of the total PLFAs) considered in the study as microbial biomarkers for the different taxonomic groups

Biomarker	Taxonomic group	References
14:0 iso, 15:0 iso, 15:0 anteiso, 15:1 iso w6c, 15:1 iso w9c, 16:0 iso, 17:0 iso, 17:0 anteiso, 17:1 iso w9c	Prokaryotes: Gram+ bacteria	Ratledge and Wilkinson (1988), Kieft et al. (1994), Paul and Clark (1996), Zelles (1999), Olsson et al. (1999), Bartelt-Ryser et al. (2005), Zelles (1999)
16:1w5c, 16:1w7c, 16:1w9c, 17:1w8c, 17:0 cyclo w7c, 18:1w5c, 18:1 w9c, 18:1w7c, 19:0 cyclo w7c	Prokaryotes: Gram− bacteria	Kieft et al. (1994), Paul and Clark (1996), Zelles (1999)
16:0 10 methyl, 17:0 10 methyl, 17:1w7c 10 methyl, 18:0 10 methyl, 18:1w7c 10 methyl	Prokaryotes: actinomycetes, Gram+ bacteria	Zelles (1999)
15:0 DMA	Prokaryotes: anaerobic bacteria	
20:4w6	Eukaryotes: protozoa	Paul and Clark (1996)
18:2w6c	Eukaryotes: fungi	Paul and Clark (1996)
16:1w5c	Eukaryotes: putative arbuscular mycorrhizas, fungi	Olson et al. (1999), Madan et al. (2002)
14:0, 15:0, 16:0, 17:0, 18:0, 20:0, 22:0, 24:0	Not assigned to a taxonomic group	Ratledge and Wilkinson (1988), Niklaus et al. (2003)

### Plant analysis

At the end of the post-flooding maize growth trial, leaf chlorophyll concentration was estimated using a SPAD 502 Portable Chlorophyll Meter (Minolta Camera Co., Osaka, Japan). In addition, leaf chlorophyll was extracted with 10 ml methanol from 1-cm^2^ pieces of young leaves (two per pot) and determined according to Wintermans and de Mots (1965). SPAD and leaf chlorophyll concentration per unit of surface were highly correlated (*r* = 0.95, *p* < 0.001); therefore, the SPAD value was adopted as a reliable proxy for chlorophyll concentration. At harvest, plant height was recorded and the roots and shoots separated and their dry weights determined by oven drying (80 °C, 48 h). Nutrient concentrations in shoots and roots were determined after dry-ashing and HCl digestion (Adrian 1973) using a 700-ES Series ICP-OES (Varian Inc., Palo Alto, CA) except C and N, using a TruSpec® analyser (Leco Corp., St Joseph, MI).

### Statistical analysis

Repeated-measures analysis of variance (rANOVA) based on a completely randomized design with four treatments (combination of factors, NR, NR + F, MR, MR + F) was performed for pH, EC, Fe, P, NH_4_^+^, NO_3_^−^, TN and DOC, in soil solution, for soil oxidation-reduction potential and for daily GHG fluxes, and with two treatments for floodwater (NR + F and MR + F. Bonferroni multiple comparison test at a probability level of 0.05 was used to detect differences between treatments. Analysis of variance (ANOVA) based on a completely randomized factorial design with two factors (flood and maize residue) was applied to cumulative GHG fluxes and PLFAs (total amount and taxonomic groups) for the flood experiment, and to SPAD, plant height, plant dry weight and total amount of nutrients in the maize shoots and roots from the pot experiment. Tukey’s test was used to find differences between factors at a probability level of 0.05. Principal component analysis (PCA) was used to explore relationships between combinations of factors and taxonomic groups (PLFAs). Additional Pearson correlations were used to explore relationships between variables. STATISTIX 10.0 software (Analytical Software, Tallahassee, FL) was used for all the analyses except for the PCA (taxonomic groups-PLFAs) which was performed in R’ with the Vegan package to include additional variables as environmental factors (daily GHG fluxes, nutrients in soil solution, redox in soil) based on multiple regression with principal components (PCs). Significance was assessed by the permutation test.

## Results

### Soil pH, electrical conductivity (EC) and soil oxidation-reduction potential

**Fig. 1:**
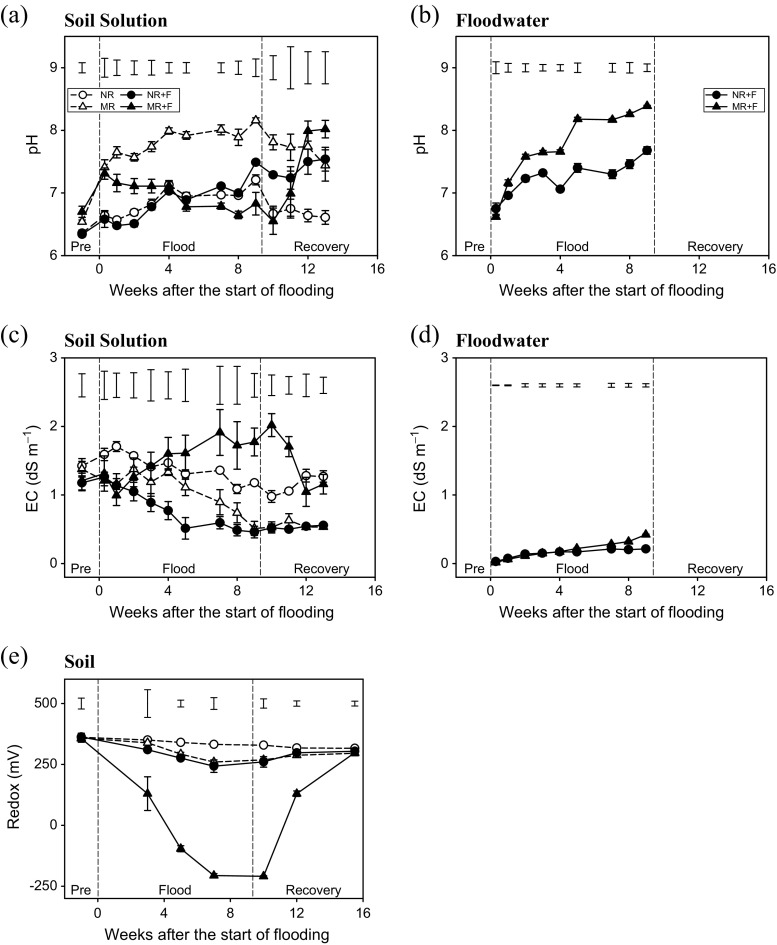
Temporal dynamics of pH (**a**, **b**), electrical conductivity (EC) (**c**, **d**) and redox potential (**e**) of soil and overlying floodwater in response to flooding and crop residue addition (8 Mg ha^−1^). Values represent means ± SEM (*n* = 4). *Vertical bars* in the upper part of the figure represent Bonferroni values for significant differences at *α* = 0.05. The *vertical lines* indicate the separation between pre-flood, flood and soil recovery phases. *NR* control without flood or residues, *NR + F* no residues with flooding, *MR* maize residue application, *MR + F* maize residue with flooding

Significant alterations in soil solution pH over the course of the experiment were observed in all treatments (*p* < 0.001 except 11 weeks after flooding when *p* = 0.444). The addition of maize residues resulted in an initial increase in soil pH, for MR during the flood phase and for MR + F at the end of the soil recovery period (Fig. 1a). A general increase in soil solution pH occurred for NR + F throughout the experiment; however, less change was seen in the NR treatment. At the end of the flood recovery phase, significant differences were still apparent between treatments. After week 1, the pH of the floodwater progressively increased over the duration of the flood for both treatments (*p* < 0.005; Fig. 1b).

Changes in soil EC in response to flooding proved highly dependent upon maize residue application, with significant effects seen within 4 weeks of flooding and persisting to the end of the soil recovery phase (*p* < 0.005). For example, EC decreased in the NR + F treatment while it increased in the MR + F treatment (Fig. 1c). In comparison to the unamended control treatment (NR), maize residue addition in the absence of flooding (MR) reduced the EC by ca. 50%. A progressive increase in floodwater EC was also observed in both flood treatments, although this was significantly greater in the presence of maize residues (*p* < 0.005; Fig. 1d).

No treatment differences in soil oxidation-reduction potential were apparent at the start of the experiment (*p* = 0.989); however, after flooding, oxidation-reduction potential dramatically reduced in the MR + F treatment relative to the other treatments. Redox potential was also slightly reduced in the MR and NR + F treatments relative to the unamended control (Fig. 1e), particularly from weeks 5 to 10 after flooding (*p* < 0.001). No significant treatment differences in soil oxidation-reduction potential were found at the end of the flood recovery phase (*p* = 0.182; Fig. 1e).

### Soil nutrient dynamics and potential losses

**Fig. 2:**
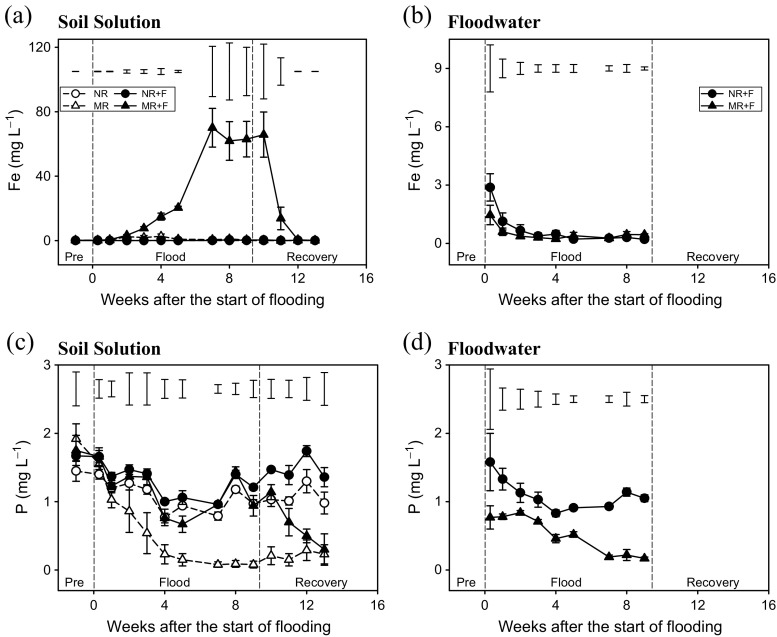
Temporal dynamics of soil solution Fe (**a**, **b**) and P (**c**, **d**) in response to flooding and crop residue addition (8 Mg ha^−1^). Values represent means ± SEM (*n* = 4). *Vertical bars* in the upper part of the figure represent Bonferroni values for significant differences at *α* = 0.05. The *vertical lines* indicate the separation between pre-flood, flood and soil recovery phases. *NR* control without flood or residues, *NR + F* no residues with flooding, *MR* maize residue application, *MR + F* maize residue with flooding

We observed the precipitation of large quantities of Fe(OH)_3_ on the container walls of all the flooded mesocosms. A very large increase in soluble Fe, particularly in the MR + F treatment, was also observed during flooding (*p* < 0.005); however, these levels rapidly fell to background within 3 weeks of floodwater removal (Fig. 2a). Initially, the floodwater contained elevated levels of Fe; however, these decreased in both treatments with increasing flood duration (Fig. 2b).

Maize residue addition progressively reduced soil solution P concentrations in the unflooded treatments. In contrast, no significant differences were observed for the NR, NR + F and MR + F treatments during flooding. In the MR + F treatment, however, removal of floodwater did stimulate a loss of P from solution. At the end of the soil recovery phase, P concentrations were significantly lower in treatments which had received maize residues, irrespective of flooding (*p* < 0.001; Fig. 2c). The concentrations of P in floodwater and soil solution were similar; however, generally smaller concentrations of P were observed in floodwater from the MR + F treatment than in the NR + F treatment (*p* < 0.05; Fig. 2d).

**Fig. 3:**
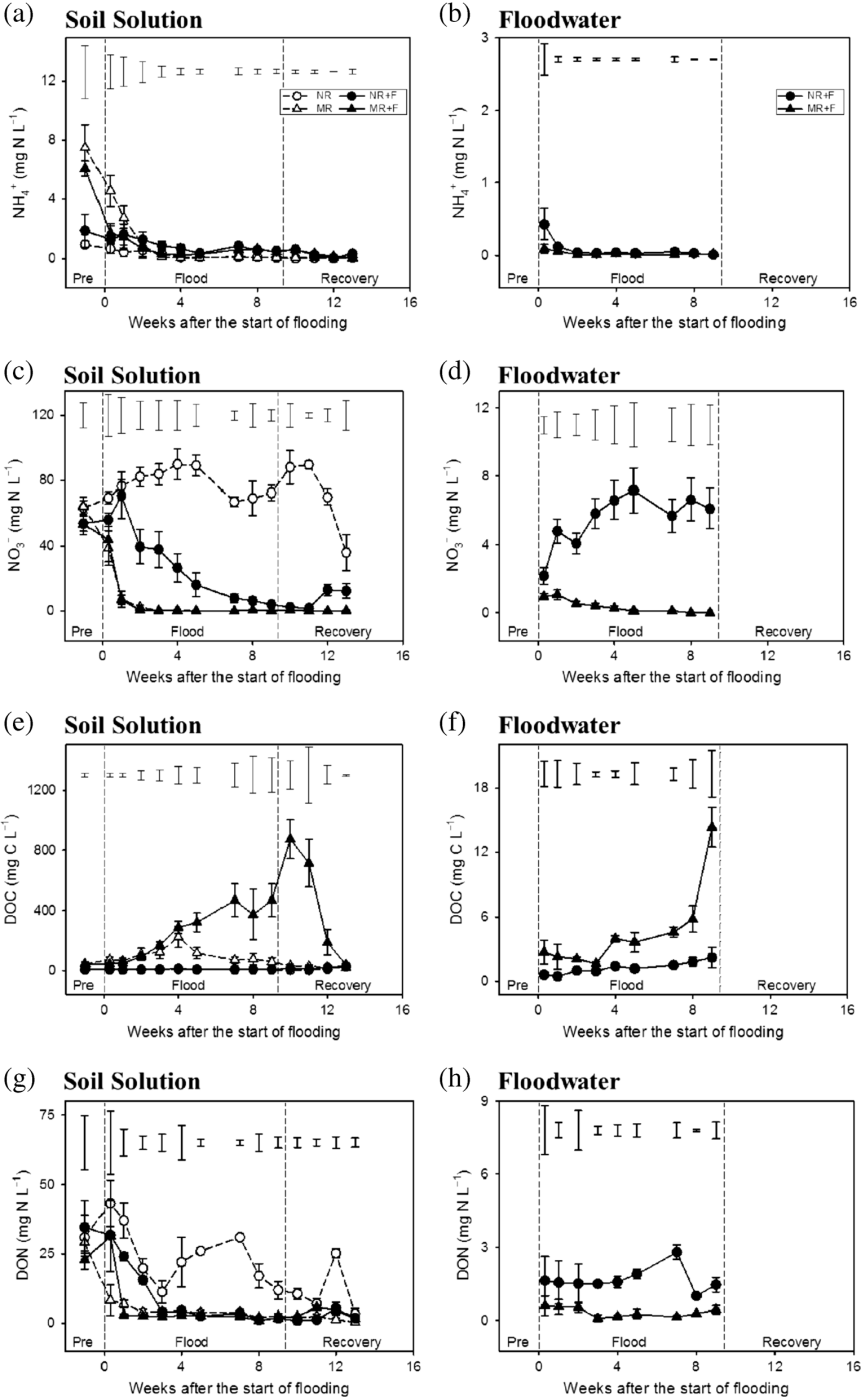
Temporal dynamics of soil solution NH_4_^+^ (**a**, **b**), NO_3_^−^ (**c**, **d**), DOC (**e**, **f**) and DON (**g**, **h**) in response to flooding and crop residue addition (8 Mg ha^−1^). Values represent means ± SEM (*n* = 4). *Vertical bars* in the upper part of the figure represent Bonferroni values for significant differences at *α* = 0.05. The *vertical lines* indicate the separation between pre-flood, flood and soil recovery phases. *NR* control without flood or residues, *NR + F* no residues with flooding, *MR* maize residue application, *MR + F* maize residue with flooding

Maize residue application initially resulted in higher soil solution NH_4_^+^ concentrations (*p* = 0.008; Fig. 3a); however, over the duration of the experiment, NH_4_^+^ levels fell in all treatments and remained low until the end of the experiment. Consistently low NH_4_^+^ concentrations (<0.5 mg N l^−1^) were also observed in the floodwater with no significant treatment effects observed (Fig. 3b). The initial soil solution concentration of NO_3_^−^ was high in all treatments with no significant treatment differences observed. However, NO_3_^−^ concentrations in the maize residue treatments quickly reached very low levels, especially in the flooded treatment (Fig. 3c). This decrease in NO_3_^−^ was also observed in the NR + F treatment during the flood phase. Similarly to soil, maize residue application reduced floodwater NO_3_^−^ concentrations relative to the unamended mesocosms. Overall, floodwater NO_3_^−^ concentrations increased in the NR + F treatment and remained low in the MR + F treatment (*p* < 0.05; Fig. 3d).

There was a large increase in soil solution DOC concentrations in the MR + F treatment relative to the MR treatment and especially in comparison to the NR and NR + F treatments. For both maize residue treatments, a reduction in DOC was observed after floodwater removal (Fig. 3e). At the end of the flood recovery phase, the concentration of DOC was similar in all treatments (*p* = 0.089). Floodwater DOC concentrations were also significantly higher in the mesocosms amended with maize residues (*p* < 0.05; Fig. 3f). The pattern of DOC and DON in the different floodwater treatments followed a similar trend to that of DOC and DON in soil solution although the concentrations were much lower (Figs. 3f–h).

Table 2 summarizes the potential losses of nutrients in response to flooding and the presence of maize crop residues. Overall, flooding caused a significant increase in potential losses for all nutrients. In addition, maize residue treatments resulted in greater potential losses of Fe, NH_4_^+^ and C but a decrease in NO_3_^−^. An interaction occurred between flood and maize residue for C with the potential losses following the order NR < NR + F < MR < MR + F.

**Table 2:** Factorial analysis of potential losses of nutrients (mean ± standard error, *n* = 8 recipients per factor, 4 replications per combination of factors) as a function of the flood and maize residue addition at the end of the experiment

Factor		Fe (kg ha^−1^)	P (kg ha^−1^)	NH_4_^+^ (kg ha^−1^)	NO_3_^−^ (kg ha^−1^)	DON (kg ha^−1^)	C (kg ha^−1^)
Flood	C	0.42 ± 0.18 b	0.36 ± 0.03 b	0.92 ± 0.33 b	16.69 ± 1.35 b	8.28 ± 1.07 b	26.5 ± 10.5 b
	F	18.58 ± 7.95 a	2.11 ± 0.27 a	2.04 ± 0.34 a	30.43 ± 3.88 a	17.85 ± 2.40 a	225.4 ± 87.5 a
*P*		**<0.001**	**<0.001**	**0.008**	**0.002**	**0.004**	**<0.001**
Maize residue	NR	1.67 ± 0.79 b	1.39 ± 0.47	0.82 ± 0.31 b	28.00 ± 4.31 a	14.80 ± 2.15	10.7 ± 2.5 b
	MR	17.33 ± 8.28 a	1.08 ± 0.26	2.14 ± 0.32 a	19.14 ± 2.49 b	11.33 ± 2.82	241.3 ± 82.5 a
*P*		**<0.001**	0.238	**0.003**	**0.027**	0.216	**<0.001**
	NR	0.08 ± 0.03	0.31 ± 0.02	0.25 ± 0.08	19.67 ± 1.03	10.36 ± 1.14	5.2 ± 0.1 d
	NR + F	3.27 ± 1.10	2.48 ± 0.49	1.39 ± 0.46	36.31 ± 6.28	19.24 ± 2.67	16.1 ± 3.0 c
	MR	0.78 ± 0.28	0.41 ± 0.05	1.60 ± 0.47	13.71 ± 1.21	6.21 ± 1.07	47.8 ± 14.8 b
	MR + F	33.89 ± 11.71	1.74 ± 0.15	2.69 ± 0.23	24.56 ± 2.79	16.46 ± 4.30	434.6 ± 81.2 a
Interaction (*P*)		0.943	0.129	0.950	0.428	0.800	**0.049**

Different letters indicate differences according to Tukey’s HSD test at a probability level of 0.05. All values for soluble N are given on an equivalent kilogram N per hectare basis. Bold indicates significant at *p* < 0.05

*C* control without flood, *F* flood, *NR* no-residue application, *MR* maize residue application (8 Mg ha^−1^)

### Soil GHG emissions

**Fig. 4:**
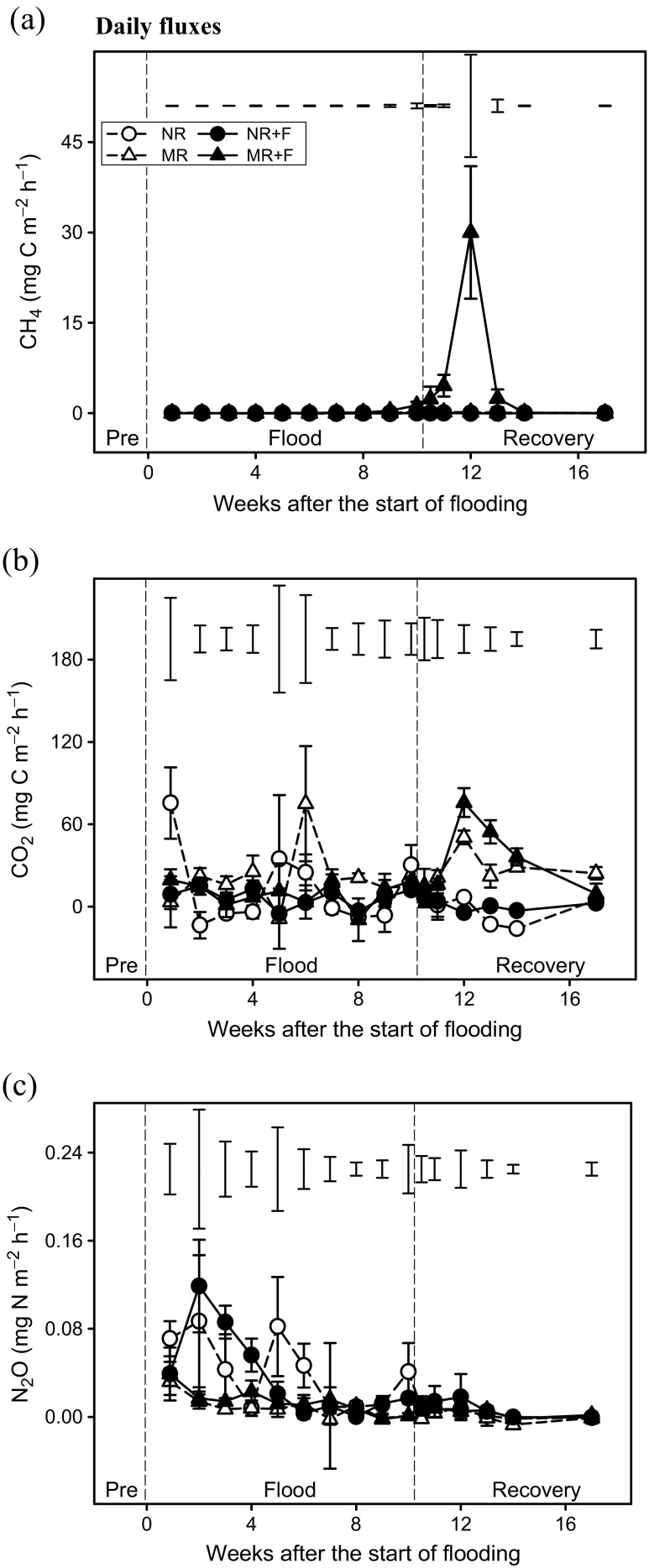
Daily fluxes of greenhouse gases (**a** CH_4_, **b** CO_2_ and **c** N_2_O) from soil in response to flooding and crop residue addition (8 Mg ha^−1^). Values represent means ± SEM (*n* = 4). *Vertical bars* in the upper part of the figure represent Bonferroni values for significant differences at *α* = 0.05. The *vertical lines* indicate the separation between pre-flood, flood and soil recovery phases. *NR* control without flood or residues, *NR + F* no residues with flooding, *MR* maize residue application, *MR + F* maize residue with flooding

Daily GHG fluxes throughout the experiment are shown in Fig. 4. Methane release was only detectable in the MR + F treatment at the end of the flood phase and during the first 4 weeks of soil recovery (Fig. 4a). In the MR treatment, peak CO_2_ emissions occurred in the first few weeks following residue addition, although this was suppressed in the presence of floodwater (Fig. 4b). In the case of N_2_O, emissions were low in comparison with the other two GHGs but some peaks were observed in the NR + F and NR treatments in the first few weeks (Fig. 4c). The overall effect of flooding and maize residue incorporation on GHG emissions and GWP is shown in Table 3. Overall, GWP followed the series

$$ \mathrm{NR}+\mathrm{F}<\mathrm{NR}<\mathrm{MR}<\mathrm{MR}+\mathrm{F}\left( p=0.060\right) $$

**Table 3:** Cumulative GHG fluxes and global warming potential (GWP, in equivalent g of CO_2_; mean ± standard error, *n* = 8 recipients per factor, 4 replications per combination of factors) as a function of flood and maize residue addition at the end of the experiment

Factor		CH_4_ kg C ha^−1^	CO_2_ kg C ha^−1^	N_2_O kg N ha^−1^	GWP kg C ha^−1^
Flood	C	0.47 ± 0.45 b	403 ± 111	0.40 ± 0.14	537 ± 105
	F	26.1 ± 14.1 a	315 ± 87	0.39 ± 0.10	1318 ± 541
*P*		**0.038**	0.232	0.943	0.081
Maize residue	NR	0.05 ± 0.23 b	123 ± 39 b	0.63 ± 011 a	313 ± 65 b
	MR	26.5 ± 14.0 a	595 ± 57 a	0.15 ± 0.03 b	1542 ± 484 a
*P*		**0.033**	**<0.001**	**0.002**	**0.011**
	NR	−0.18 ± 0.34 b	148 ± 77	0.69 ± 0.18	348 ± 124 b
	NR + F	0.29 ± 0.31 b	98 ± 29	0.57 ± 0.15	277 ± 61 b
	MR	1.13 ± 0.73 b	658 ± 95	0.10 ± 0.04	726 ± 111 ab
	MR + F	51.9 ± 21.9 a	531 ± 61	0.21 ± 0.03	2358 ± 800 a
Interaction (*P*)		**0.041**	0.598	0.363	0.060

Different letters indicate differences according to Tukey’s HSD test at a probability level of 0.05. GWP for CH_4_ is 34, and that for N_2_O is 298 (IPCC, 2013). Bold indicates significant at *p* < 0.05

*C* control without flood, *F* flood, *NR* no-residue application, *MR* maize residue application (8 Mg ha^−1^)

Table S1 displays the correlations between soil parameters and GHG fluxes during the flood and soil recovery stages. Of notable importance are the relationships between oxidation-reduction potential and nutrient release (Fe, NH_4_^+^, DOC) and GHG fluxes (CH_4_ and CO_2_) during flooding and with N_2_O in the flood recovery phase. In addition, there were strong relationships between pH and nutrient concentration (EC, Fe, P, NO_3_^−^) during flooding.

### Soil microbial community composition

The two factors, flood and maize residue, increased microbial biomass (total PLFA) at the two sampling times (Table 4). These differences were driven by an increase in the amount of Gram− bacteria and anaerobic bacteria (anaerobes); however, actinomycetes and fungi were both reduced by flooding. Gram− bacteria, anaerobes, protozoa and fungi (after the flood phase) were positively affected, actinomycetes negatively (both samplings), and putative arbuscular mycorrhiza (AM) decreased during flooding but increased during the soil recovery phase in the presence of maize residues (Table 4). Total microbial biomass (PLFAs) and the majority of individual fatty acids were positively correlated with EC, Fe, DOC, CH_4_ and CO_2_ when analysed at the end of the flood phase and pH after the soil recovery, but negatively with soil oxidation-reduction potential and NO_3_^−^ (both samplings), P and N_2_O after the soil recovery phase (Table S2).

**Table 4:** Total amount of PLFAs (nmol g^−1^) and taxonomic groups (%; mean ± standard error, *n* = 8 recipients per factor, 4 replications per combination of factors) as a function of flood and maize residue addition at the end of the experiment

		Microbial PLFAs (nmol g^−1^)	Gram+ bacteria (%)	Gram– bacteria (%)	Actinomycetes (%)	Anaerobes (%)	Protozoa (%)	Arb. myco. (%)	Fungi %
After flood stage									
Flood	C	16.7 ± 1.0 b	31.0 ± 0.5	44.2 ± 1.0 b	17.1 ± 0.8 a	1.4 ± 0.2 b	0.5 ± 0.1	4.1 ± 0.1	1.8 ± 0.1 a
	F	20.4 ± 2.3 a	30.7 ± 0.9	46.0 ± 1.4 a	15.5 ± 1.3 b	1.8 ± 0.1 a	0.6 ± 0.2	3.9 ± 0.1	1.5 ± 0.1 b
*P*		**0.022**	0.542	**0.003**	**< 0.001**	**0.003**	0.305	0.087	**0.018**
Maize residue	NR	14.5 ± 0.3 b	32.5 ± 0.3 a	41.2 ± 0.3 b	19.0 ± 0.1 a	1.3 ± 0.1 b	0.3 ± 0.2 b	4.2 ± 0.1 a	1.5 ± 0.1 b
	MR	22.7 ± 1.5 a	29.2 ± 0.5 b	49.0 ± 0.9 a	13.5 ± 0.6 b	1.9 ± 0.1 a	0.8 ± 0.1 a	3.8 ± 0.1 b	1.8 ± 0.1 a
*P*		**<0.001**	**<0.001**	**<0.001**	**<0.001**	**<0.001**	**0.011**	**<0.001**	**0.024**
	NR	14.3 ± 0.6 c	32.1 ± 0.4 ab	41.6 ± 0.4 c	19.3 ± 0.1 a	1.0 ± 0.1 c	0.1 ± 0.1	4.2 ± 0.1 a	1.6 ± 0.1 ab
	MR	19.1 ± 0.6 b	29.9 ± 0.4 bc	46.7 ± 0.5 b	14.9 ± 0.3 b	1.8 ± 0.1 a	0.8 ± 0.1	4.0 ± 0.1 ab	2.0 ± 0.1 a
	NR + F	14.6 ± 0.3 c	32.8 ± 0.4 a	40.9 ± 0.4 c	18.8 ± 0.2 a	1.5 ± 0.1 b	0.4 ± 0.3	4.2 ± 0.1 a	1.4 ± 0.1 b
	MR + F	26.2 ± 1.4 a	28.5 ± 0.9 c	51.2 ± 0.7 a	12.1 ± 0.4 c	2.0 ± 0.1 a	0.8 ± 0.1	3.7 ± 0.1 b	1.6 ± 0.2 ab
Interaction (*P*)		0.123	0.093	**<0.001**	**0.001**	0.161	0.520	0.199	0.386
After soil recovery									
Flood	C	22.9 ± 1.7	26.1 ± 0.4	43.9 ± 1.3	15.1 ± 0.6	3.7 ± 0.7	2.6 ± 0.3	4.0 ± 0.1	4.6 ± 0.3
	F	26.8 ± 2.1	26.2 ± 0.9	45.3 ± 1.1	14.7 ± 0.7	2.9 ± 0.5	3.0 ± 0.8	3.7 ± 0.1	4.2 ± 0.7
*P*		0.052	0.975	0.116	0.600	0.367	0.602	0.065	0.576
Maize residue	NR	21.0 ± 1.0 b	26.5 ± 0.9	41.7 ± 0.5 b	16.3 ± 0.5 a	3.9 ± 0.7	3.6 ± 0.7	3.7 ± 0.1 b	4.3 ± 0.6
	MR	28.7 ± 1.7 a	25.8 ± 0.4	47.5 ± 0.7 a	13.5 ± 0.4 b	2.7 ± 0.4	1.9 ± 0.1	4.0 ± 0.1 a	4.5 ± 0.4
*P*		**0.001**	0.550	**<0.001**	**0.002**	0.135	0.054	**0.023**	0.733
	NR	19.6 ± 0.4 b	26.7 ± 0.7	40.9 ± 0.6 b	16.5 ± 0.4 a	5.1 ± 1.0	3.1 ± 0.4	3.7 ± 0.1 ab	4.0 ± 0.4
	NR + F	22.4 ± 1.8 b	26.3 ± 1.8	42.5 ± 0.6 b	16.1 ± 1.1 ab	2.8 ± 0.8	4.2 ± 1.5	3.7 ± 0.2 b	4.6 ± 1.3
	MR	26.2 ± 2.4 ab	25.6 ± 0.5	46.9 ± 1.0 a	13.7 ± 0.6 ab	2.3 ± 0.4	2.1 ± 0.2	4.3 ± 0.1 a	5.2 ± 0.1
	MR + F	31.3 ± 2.1 a	26.0 ± 0.7	48.0 ± 0.9 a	13.4 ± 0.5 b	3.1 ± 0.8	1.8 ± 0.1	3.8 ± 0.1 ab	3.9 ± 0.5
Interaction (*P*)		0.539	0.696	0.756	0.888	0.071	0.424	0.157	0.214

Bold indicates significant at *p* < 0.05

*C* control without flood, *F* flood, *NR* non-residue application, *MR* maize residue application (8 Mg ha^−1^), *Anaerobes* anaerobic bacteria, *Arb. Myco.* putative arbuscular mycorrhizal fungi

**Fig. 5:**
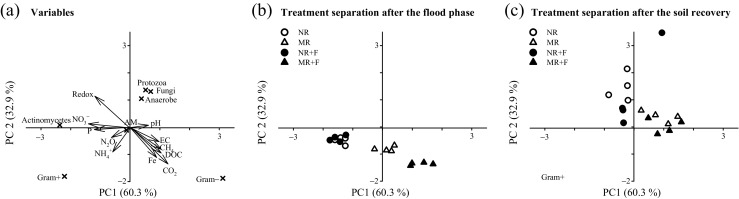
Principal component analysis of microbial community PLFAs in response to flooding and crop residue addition (8 Mg ha^−1^). **a** Relationships between taxonomic groups (PLFAs, *crosses*, used to separate the different combination of treatments) and environmental variables (*arrows*, redox in soil, pH, EC, P, Fe, NH_4_^+^, NO_3_^−^, DOC, CH_4_, CO_2_, N_2_O, showing their relations with different taxonomic groups); **b** treatment separation after the flood phase; and **c** treatment separation after the soil recovery phase. *NR* control without flood or residues, *NR + F* no residues with flooding, *MR* maize residue application, *MR + F* maize residue with flooding

Figure 5 shows the PCA for the taxonomic groups (PLFAs) and their relationship to environmental variables. The first PC, which explained 60.3% of total variance, is related with abundances of Gram− bacteria in one direction and abundances of actinomycetes and Gram+ bacteria in the opposite direction. The second PC, which explained 32.9% of the variance, is related to abundances of protozoa, fungi and anaerobe in one direction and abundances of Gram− and Gram+ bacteria in the other. The variables most strongly related to the first two PCs are oxidation-reduction potential (*p* < 0.001, *r* ^2^ = 0.47), CO_2_ (*p* = 0.002, *r* ^2^ = 0.54), DOC (*p* = 0.003, *r* ^2^ = 0.34), NO_3_^−^ (*p* = 0.006, *r* ^2^ = 0.30), CH_4_ (*p* = 0.028, *r* ^2^ = 0.28), Fe (*p* = 0.017, *r* ^2^ = 0.27), P (*p* = 0.017, *r* ^2^ = 0.24) and EC (*p* = 0.042, *r* ^2^ = 0.20) (Fig. 5a). The PCA was able to clearly separate the soil treatments by time along PC1. Soil samples collected after the flood phase were below the *x*-axes (Fig. 5b), but the majority of those collected after the soil recovery phase were located above (Fig. 5c).

### Post-flooding maize growth trial

At the final harvest, plant dry weight followed the series NR > MR + F > MR > NR + F (*p* = 0.050; Table 5). The lowest SPAD values were obtained for plants grown on the NR + F soil samples. Overall, maize residue application reduced the N content of the shoots and roots (*p* = 0.018 and *p* = 0.001, Table S3) while increasing the shoot-to-root ratio (*p* = 0.013). In contrast, flooding appeared to have little effect on plant performance or nutrient content (Table 5, Table S3).

**Table 5:** Effect of flooding and soil maize residues on plant height, leaf chlorophyll content (SPAD) and plant biomass parameters of maize plants grown in the different soil samples at the end of the flood recovery phase (4-week length). The experimental unit was a pot with two maize plants

Factor		Height (cm)	SPAD values	Shoot dry weight (g)	Root dry weight (g)	Plant dry weight (g)	Ratio above ground-biomass/root
Flood	C	56.6 ± 1.0	16.2 ± 1.3	2.45 ± 0.16	1.34 ± 0.12	3.78 ± 0.25	1.9 ± 0.1
	F	54.3 ± 1.9	14.9 ± 1.6	2.30 ± 0.17	1.26 ± 0.08	3.55 ± 0.21	1.8 ± 0.1
*P*		0.184	0.386	0.498	0.531	0.452	0.833
Maize residue	NR	53.3 ± 1.7 b	13.2 ± 1.4 b	2.29 ± 0.20	1.41 ± 0.12	3.67 ± 0.30	1.6 ± 0.1 b
	MR	57.5 ± 1.0 a	18.0 ± 0.9 a	2.46 ± 0.12	1.20 ± 0.06	3.65 ± 0.16	2.1 ± 0.1 a
*P*		**0.028**	**0.006**	0.431	0.128	0.948	**0.013**
	NR	56.4 ± 1.3 ab	15.5 ± 2.2 ab	2.58 ± 0.31	1.56 ± 0.16	4.11 ± 0.42	1.7 ± 0.2
	NR + F	50.3 ± 2.3 b	10.9 ± 0.7 b	2.00 ± 0.19	1.25 ± 0.16	3.23 ± 0.31	1.6 ± 0.1
	MR	56.8 ± 1.8 ab	16.9 ± 1.6 a	2.33 ± 0.13	1.13 ± 0.09	3.45 ± 0.21	2.1 ± 0.1
	MR + F	58.3 ± 0.8 a	19.0 ± 0.6 a	2.60 ± 0.20	1.27 ± 0.07	3.86 ± 0.21	2.1 ± 0.2
Interaction (*P*)		**0.039**	**0.038**	0.071	0.101	**0.050**	0.919

Different letters indicate significant differences determined using Tukey HSD post hoc tests at the 0.05 probability level. Bold indicates significant at *p* < 0.05

*C* control without flood, *F* flood, *NR* non-residue application, *MR* maize residue application (8 Mg ha^−1^)

## Discussion

### Soil solution chemistry

Here, we show that the chemistry of an arable soil with no known history of flooding responds very quickly to extreme waterlogging. Of importance, however, is that almost all of the soil chemical quality indicators studied showed reversible changes, returning back to levels seen in the corresponding control treatments within weeks of floodwater removal. Our study therefore demonstrates the resilience of soil to extreme winter flooding events, although this should be extrapolated to other soil types with caution (Hueso et al. 2011; de Vries et al. 2012). We further conclude that the presence of cereal residues in combination with flooding appears to be far more influential in regulating soil chemistry than flooding alone. Lastly, the soil chemical responses to waterlogging seen in our soil mirrored those frequently seen in soils with a long history of waterlogging (e.g. Gleysols, Fluvisols; Kyuma 2004; Rinklebe et al. 2016), suggesting that existing information on hydromorphic soils may be useful in predicting the impact of extreme flooding in soils rarely exposed to flooding.

Flooding did, however, increase the potential for nutrient losses (e.g. NO_3_^−^, DON, P, C). In addition, flooding caused a redistribution of Fe within the soil. At the end of the flood period, it was apparent that Fe had migrated from the anoxic subsurface layers and re-precipitated as Fe-oxyhydroxides at the soil surface. This was most apparent in the residue treatments where the presence of labile C fuels a drop in oxidation-reduction potential and the reduction and release of Fe^2+^ (Gotoh and Patrick 1974). We had expected that P would be released from the surface of Fe minerals when they were reduced; however, there was no evidence to support this. We speculate that any released P was either re-sorbed onto Al(OH)_3_, migrated to the soil surface where it re-precipitated with Fe or was immobilized by the growing microbial community (Bünemann et al. 2012; Damon et al. 2014). All of these may have implications for future fertilizer management and plant P availability (Kögel-Knabner et al. 2010).

### Soil greenhouse gas emissions

Consistent with previous studies, the presence of maize residues and flooding caused major changes in net GHG emissions (Smith and Conen 2004); however, these effects did not persist long into the soil recovery phase. The greatest emissions occurred in the flooded treatments when crop residues were present. We ascribe this to the microbial use of the added C, the consumption of O_2_ and a progressive drop in oxidation-reduction potential that ultimately resulted in CH_4_ production (Hou et al. 2000). Maximal CH_4_ release occurred after floodwater removal probably from the release of CH_4_ trapped within the soil pores (Comas and Wright 2012). It is also likely that the abundance of alternative terminal electron acceptors (e.g. NO_3_^−^ ➔ Fe^3+^ ➔ Mn^4+^ ➔ SO_4_^2−^) limited CH_4_ production in the first 6–8 weeks of flooding (Cheng et al. 2006). This is supported by the dynamics of NO_3_^−^ loss, Fe^2+^ production and the visual presence of FeS around the crop residues. Our results therefore suggest that shorter periods of soil inundation (<4 weeks) with no history of flooding should result in very low CH_4_ emissions, possibly due to the lack of a large methanogen community (Hansen et al. 2014). CH_4_ consumption can form an important part of the CH_4_ budget in some soils (Liu et al. 2016). As we did not directly evaluate this process, further work is required to clarify if methanotrophy is affected following floodwater removal. Flooding caused a major loss of soil NO_3_^−^; however, this did not translate into increased N_2_O emissions, presumably due to complete denitrification (to N_2_) or immobilization within the microbial biomass (Baggs et al. 2000; Chen et al. 2013; Li et al. 2013). This is consistent with the lack of O_2_, the high C:N ratio of our crop residues (Gentile et al. 2008) and the low NH_4_^+^ concentrations in our soil solution (Rice and Tiedje 1989). N_2_O emissions were greatest in the absence of crop residues. In the unflooded treatment, this could arise from the simultaneous operation of nitrification and denitrification processes within the soil matrix (Gentile 2008; Bateman and Baggs 2005; Butterbach-Bahl et al. 2013), while under flooding, we expect that denitrification dominates (Amend and Shock 2001).

Flooding has been shown to either promote or reduce CO_2_ emissions depending upon land management regime (Aulakh et al. 2001; Li et al. 2004; Loeb et al. 2008). Although a reduction in the production of CO_2_ was expected due to the increase in the salinity of soil solution observed when the flooded soil was amended with maize residues (high ratio C/N; Hasbullah and Marschner 2015), in our experiment, inundation caused little change in net CO_2_ emissions. The similar rates of CO_2_ production at the end of the experiment are also consistent with a lack of effect of flooding on soil microbial function. Studies in which rice straw was incorporated into the soil (Tang et al. 2016) have shown that the production of CH_4_ and CO_2_ under anaerobic conditions were lowest at higher temperatures because the residues were highly decomposed due to the previous aerobic conditions. Therefore, as our experiment was developed under a simulated spring flood event, the production of these two gases was expected. The results presented here also suggest that long-term flooding of low-lying agricultural areas should be accounted for within national GHG inventory reporting.

### Soil microbial community biomass and composition

Although not observed in many previous studies involving crop residues, the presence of maize stover increased the size of the microbial biomass and also induced a shift in community composition (Hoyle and Murphy 2006; Sall et al. 2015; Zhao et al. 2016). We ascribe this growth to the high relative abundance of N and P and the removal of C limitation within the system (Cayuela et al. 2009). These microbial community shifts were further enhanced by flooding. Our results are in partial agreement with Bossio and Scow (1995, 1998) and Unger et al. (2009) who showed little effect of flooding on the microbial biomass, but that it did induce shifts in the PLFA profile. Consistent with the reduction in oxidation-reduction potential, release of Fe^2+^ and production of CH_4_, we showed an increase in the PLFA marker for anaerobic bacteria and a reduction in obligate aerobes in the flooded treatments (Reichardt et al. 2001). The increase in DOC in the flooded treatments is also consistent with the lack of O_2_ required to promote enzymes involved in lignin breakdown (e.g. phenol oxidase) and the production of humic-rich DOC. The effects of flooding largely disappeared after the soil recovery phase. This is consistent with the other soil quality indicator values returning back to those seen in the unflooded controls. Although we cannot say whether flooding induced the loss of individual species, the high degree of functional redundancy within the community and the lack of major changes in PLFA profiles suggest that flooding did not cause irreversible changes in the community. Further work is required, however, to look at potential changes in keystone species involved in nutrient cycling (e.g. N-fixers, nitrifiers, denitrifiers, mycorrhizal fungi) and the potential changes in plant pests and diseases (e.g. nematodes, damping-off).

### Plant performance

Here, we show that in comparison to the presence or absence of crop residues, maize growth was not greatly influenced by flood history. This supports field observations that a single flood event does not cause an irreversible loss in soil function and its ability to support plant growth, at least in the short term (Defra 2014). Outside of rice cropping systems, however, the longer-term post-consequences of extreme flood events on crop growth still remain poorly understood.

The period of recovery used here was designed to simulate the earliest time at which farmers would intervene after floodwater removal (based on field observation). This seems appropriate given that most soil quality indicators (e.g. GHG emissions, DOC, Fe, P, oxidation-reduction potential) had returned to levels close to the unflooded controls by 5–8 weeks. Our results also suggest that any phytotoxic substances created under flooding had either been biodegraded (e.g. volatile fatty acids), oxidation-reduction potential-sensitive metals had re-precipitated (e.g. Mn^2+^ ➔ MnO_2_), or that they had become re-adsorbed to the solid phase (e.g. Zn; Du Laing et al. 2009). This is also supported by the lack of observed effect of flooding on root or foliar metal content. In our experience, farmers may wait up to 9 months before attempting to re-establish a crop on land that has been unduly flooded, due to the risk of causing compaction when tilling a wet soil. Under these circumstances, it is likely that all traces of phytotoxins will have disappeared from the soil (Chou and Lin 1976).

### Conclusions and implications for management

As expected, extreme flooding had a major impact, albeit short term, on soil functioning. Further, the evidence presented here suggests that long-term flooding in the presence of crop residues may have more severe consequences, in terms of soil quality, than if residues are not present. This is also supported by our own empirical field observations. A key question is therefore whether we should alter land management practices in high-flood-risk areas to limit their detrimental impact. By their nature, extreme flooding events are almost impossible to predict. In addition, it is notoriously difficult to communicate the risks of extreme weather events to farmers and persuade them to alter their land management practices (Haigh et al. 2015; Hyland et al. 2016). One potential option is that farmers in high-flood-risk areas should not incorporate crop residues into the soil or that they should be removed at harvest (e.g. by burning). While theoretically possible, this option directly contravenes current advice, albeit controversial (Mitchell et al. 2016), to incorporate residues to build up soil organic matter reserves and improve general soil health. Residue removal could also be environmentally damaging and leave the soil more prone to both water and wind erosion (Xin et al. 2016). A more radical option would be to prevent cereal cropping in high-flood-risk areas and shift to a less flood-sensitive land use (e.g. permanent grassland with high diversity; Natural England 2014; Wright et al. 2017). As with most land management options, however, there are trade-offs and the positive aspects should also be emphasized. While residues increase GHG emissions tenfold on a GWP basis, they may be beneficial in reducing NO_3_^−^ leaching and potentially indirect N_2_O emissions, both of which are universal goals for sustainable agriculture.

The work undertaken here was limited to one soil type studied under very controlled conditions in the laboratory. Further work should therefore investigate a greater range of soil types and flood scenarios, especially under realistic field conditions. In addition, studies should also include a wider range of organic residues (e.g. differing in C-to-N ratio) as this is likely to have a major impact on net GHG emissions and other soil quality indicators. Finally, as green cover crops offer great potential to protect soils from intense rainfall and flood events (Boardman 2015), further work should investigate whether their benefits exist further than just limiting erosion or whether in fact they further exacerbate the negative consequences of flooding on soil health.

## Electronic supplementary material

ESM 1 (DOCX 5002 kb)
