# Supplementary material for: Crop residues exacerbate the negative effects of extreme flooding on soil quality
Source: Biol Fertil Soils. 2017 Jun 19;53(7):751–65. doi: 10.1007/s00374-017-1214-0 (PMC6961515; doi:10.1007/s00374-017-1214-0)
Supplement: Supplementary file 1 — (DOCX 5002 kb) [file 374_2017_1214_MOESM1_ESM.docx]

**On-line Supplementary Information**

**Crop residues exacerbate the negative effects of extreme flooding on soil quality**

Antonio Rafael Sánchez-Rodríguez, Paul W. Hill, David R. Chadwick and Davey L. Jones *School of the Environment, Natural Resources and Geography, College of Natural Sciences, Bangor University, Gwynedd LL57 2UW, UK*


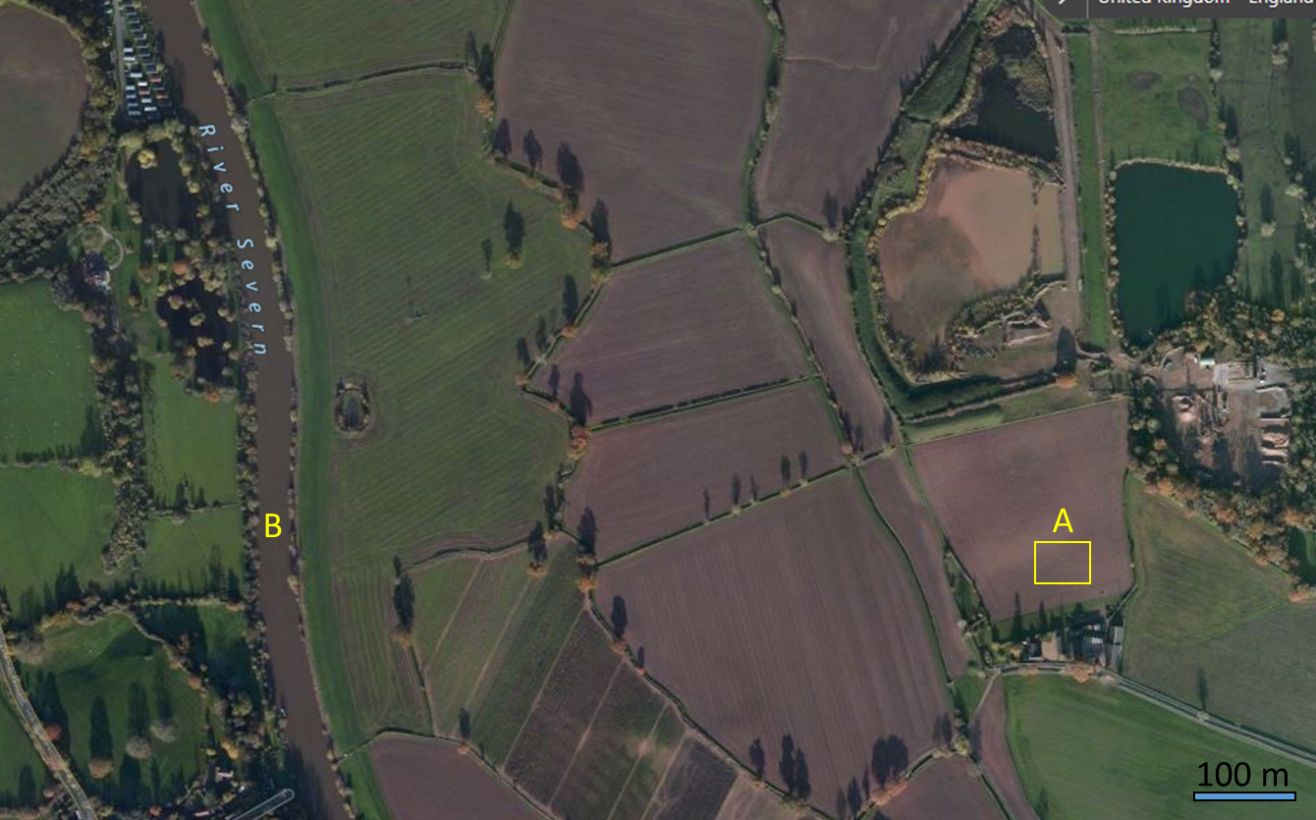


**Fig. S1** Aerial photograph showing (A) the field used for soil collection, and (B) the location of the River Severn which regularly floods the fields closest to the river.


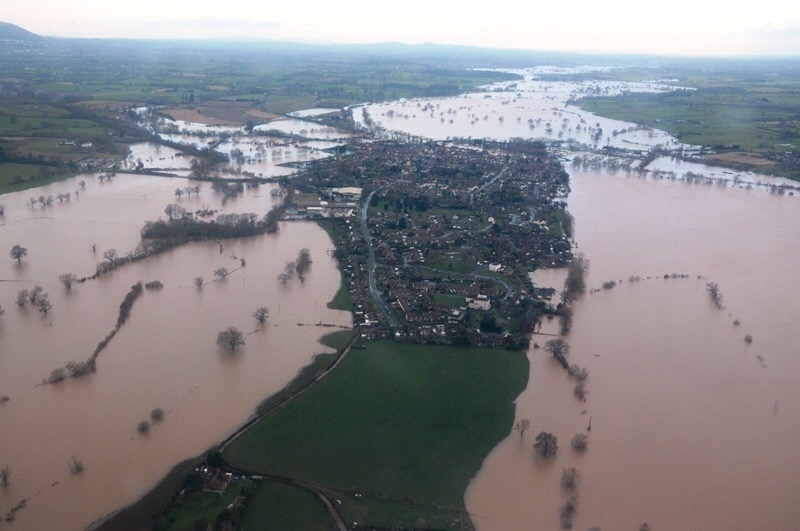

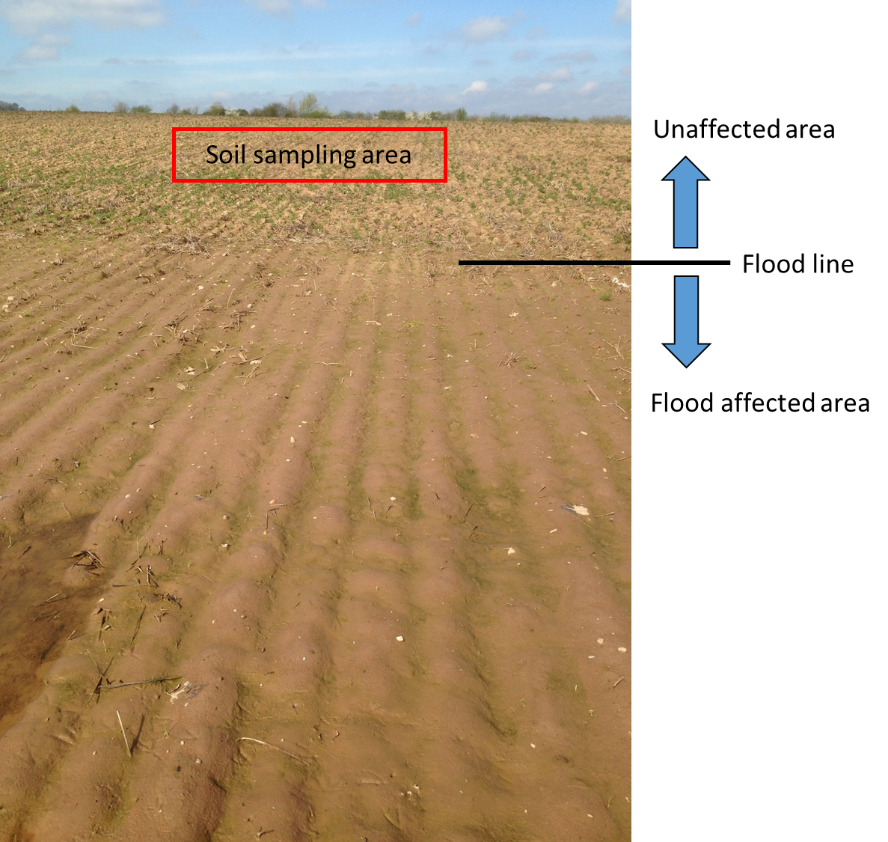


**A**

**B**

**Fig. S2** **(A)** Aerial photograph showing flooding in 2014 within the River Severn region. The soil was collected from a field above the flood line. Image courtesy of Dave Throup, Environment Agency Manager for Herefordshire and Worcestershire, UK. **(B)** Photograph showing the area where the soil used in the experiments was collected.


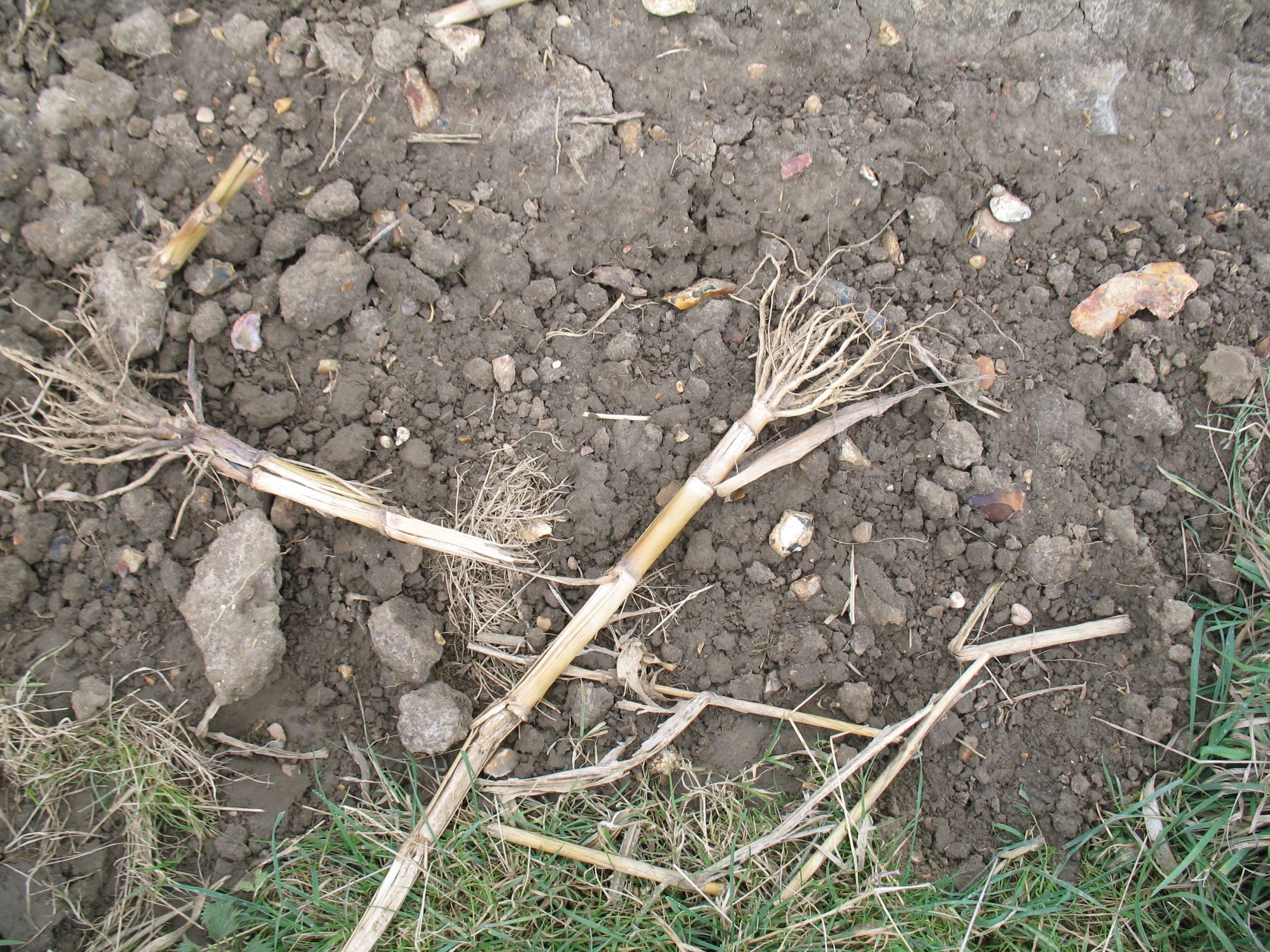


**Fig. S3** Example of the maize residues collected from the field and used in the experiments.

| **Table S1** Correlation matrix showing the relationship between GHG emissions and soil parameters after flooding and after a period of soil recovery. Significant relationships are denoted in bold. *, ** and *** indicate significant relationships at the *p* < 0.05, *p* < 0.01 and p <0.001 level respectively. | | | | | | | | | | | |
| --- | --- | --- | --- | --- | --- | --- | --- | --- | --- | --- | --- |
| After flood stage | pH | EC | Redox | Fe | P | NH_4_^+^ | NO_3_^−^ | TC | CH_4_ | CO_2_ | N_2_O |
| pH |  | **−0.76^***^** | **0.47^*^** | **−0.45^*^** | **−0.49^*^** | −0.22 | **−0.44^*^** | −0.36 | −0.15 | −0.09 | −0.11 |
| EC |  |  | **−0.85^***^** | **0.73^***^** | 0.23 | 0.34 | −0.02 | **0.74^**^** | **0.48^*^** | **0.62^**^** | 0.07 |
| Redox |  |  |  | **−0.76^***^** | −0.19 | **−0.54^*^** | 0.44^*^ | **−0.79^***^** | **−0.68^**^** | **−0.73^**^** | −0.20 |
| Fe |  |  |  |  | 0.11 | **0.42^*^** | −0.26 | **0.97^***^** | 0.38 | 0.57^*^ | 0.18 |
| P |  |  |  |  |  | **0.60^**^** | 0.10 | 0.03 | 0.12 | -0.33 | 0.13 |
| NH_4_^+^ |  |  |  |  |  |  | −0.55^*^ | 0.40 | 0.27 | 0.11 | 0.30 |
| NO_3_^−^ |  |  |  |  |  |  |  | −0.31 | −0.23 | **−0.44^*^** | −0.28 |
| TC |  |  |  |  |  |  |  |  | **0.52^*^** | **0.61^**^** | 0.17 |
| CH_4_ |  |  |  |  |  |  |  |  |  | 0.35 | 0.15 |
| CO_2_ |  |  |  |  |  |  |  |  |  |  | 0.01 |
| N_2_O |  |  |  |  |  |  |  |  |  |  |  |
| After soil recovery | pH | EC | Redox | Fe | P | NH_4_^+^ | NO_3_^−^ | TC | CH_4_ | CO_2_ | N_2_O |
| pH |  | −0.29 | −0.34 | 0.27 | −0.33 | −0.17 | **−0.49^*^** | 0.25 | 0.16 | 0.09 | −0.24 |
| EC |  |  | 0.21 | 0.08 | −0.14 | −0.26 | **0.43^*^** | 0.16 | −0.38 | −0.26 | −0.19 |
| Redox |  |  |  | −0.27 | 0.28 | 0.09 | **0.45^*^** | −0.14 | 0.02 | 0.07 | **0.53^*^** |
| Fe |  |  |  |  | 0.05 | 0.00 | −0.22 | **0.48^*^** | −0.29 | −0.10 | −0.08 |
| P |  |  |  |  |  | 0.40 | 0.29 | 0.24 | 0.25 | **−0.64^***^** | 0.40 |
| NH_4_^+^ |  |  |  |  |  |  | −0.10 | **0.59^**^** | −0.20 | −0.14 | 0.18 |
| NO_3_^−^ |  |  |  |  |  |  |  | −0.15 | 0.02 | −0.31 | 0.21 |
| TC |  |  |  |  |  |  |  |  | −0.28 | **−0.43^*^** | −0.17 |
| CH_4_ |  |  |  |  |  |  |  |  |  | 0.08 | 0.12 |
| CO_2_ |  |  |  |  |  |  |  |  |  |  | 0.11 |
| N_2_O |  |  |  |  |  |  |  |  |  |  |  |

| **Table S2** Correlation matrix between fatty acids whose amount was higher than 2 % of the total PLFAs and GHG daily fluxes (only after flood stage), redox potential in soil, NH_4_^+^, NO_3_^−^, DON and DOC). Significant relationships are denoted in bold. *, ** and *** indicate significant relationships at the *p* < 0.05, *p* < 0.01 and *p* <0.001 level, respectively. | | | | | | | | | | | | | | |
| --- | --- | --- | --- | --- | --- | --- | --- | --- | --- | --- | --- | --- | --- | --- |
|  | Biomass (PLFAs) | 15:0 iso | 15:0 anteiso | 16:0 iso | 16:1 w7c | 16:1 w5c | 16:0 | 16:0  10 methyl | 17:0  cyclo w7c | 18:2 w6c | 18:1 w9c | 18:1 w7c | 18:0 | 19:0  cyclo w7c |
|  | **After flood stage** | | | | | | | | | | | | | |
| pH | −0.21 | −0.15 | −0.27 | −0.20 | −0.244 | −0.18 | −0.19 | −0.20 | 0.12 | −0.02 | −0.10 | −0.255 | −0.33 | −0.11 |
| EC | **0.72^**^** | **0.67^**^** | **0.73^**^** | **0.66^**^** | **0.75^**^** | **0.69^**^** | **0.70^**^** | **0.59^*^** | 0.47 | 0.49 | **0.59^*^** | **0.75^**^** | **0.62^*^** | 0.36 |
| Redox | **−0.90^***^** | **−0.87^***^** | **−0.88^***^** | **−0.86^***^** | **−0.92^***^** | **−0.87^***^** | **−0.88^***^** | **−0.72^**^** | **−0.70^**^** | **−0.62^*^** | **−0.78^**^** | **−0.91^***^** | **−0.66^**^** | −0.43 |
| Fe | **0.76^**^** | **0.75^**^** | **0.84^***^** | **0.81^***^** | **0.71^**^** | **0.74^**^** | **0.78^***^** | **0.58^*^** | **0.56^*^** | **0.71^**^** | **0.76^**^** | **0.76^**^** | **0.65^**^** | 0.48 |
| P | −0.16 | −0.21 | −0.21 | 0.05 | −0.10 | −0.14 | −0.20 | −0.18 | −0.14 | −0.49 | −0.44 | −0.27 | −0.07 | 0.01 |
| NH_4_^+^ | 0.34 | 0.33 | 0.40 | 0.43 | 0.33 | 0.31 | 0.34 | 0.28 | 0.12 | 0.09 | 0.26 | 0.37 | 0.38 | 0.25 |
| NO_3_^−^ | −0.49 | **−0.54^*^** | −0.47 | **−0.52^*^** | −0.47 | −0.48 | **−0.51^*^** | −0.35 | −0.57^*^ | −0.44 | **−0.50^*^** | −0.45 | −0.29 | −0.17 |
| DOC | **0.84^***^** | **0.83^***^** | **0.86^***^** | **0.86^***^** | **0.79^***^** | **0.83^***^** | **0.85^***^** | **0.71^**^** | **0.68^**^** | **0.74^**^** | **0.83^***^** | **0.85^***^** | **0.74^**^** | **0.58^*^** |
| CH_4_ | **0.73^**^** | **0.70^**^** | **0.53^*^** | **0.61^*^** | **0.75^**^** | **0.75^**^** | **0.67^**^** | **0.78^***^** | **0.61^*^** | **0.35** | **0.66^**^** | **0.78^***^** | **0.64^**^** | **0.64^**^** |
| CO_2_ | **0.81^***^** | **0.80^***^** | **0.86^***^** | **0.78^***^** | **0.82^***^** | **0.77^**^** | **0.82^***^** | 0.46 | **0.80^***^** | **0.83^***^** | **0.75^**^** | **0.76^**^** | 0.34 | 0.15 |
| N_2_O | 0.19 | 0.25 | 0.13 | 0.32 | 0.15 | 0.16 | 0.20 | 0.38 | 0.15 | 0.00 | 0.20 | 0.17 | 0.47 | 0.39 |
|  | **After soil recovery stage** | | | | | | | | | | | | | |
| pH | **0.68^**^** | **0.75^**^** | **0.72^**^** | **0.76^**^** | **0.64^**^** | **0.64^**^** | **0.55^*^** | **0.66^**^** | **0.65^**^** | 0.44 | **0.61^*^** | **0.58^*^** | **0.60^*^** | **0.67^**^** |
| EC | −0.01 | 0.01 | 0.09 | 0.07 | 0.05 | −0.07 | −0.12 | 0.20 | 0.11 | −0.37 | 0.09 | 0.00 | −0.15 | −0.11 |
| Redox | −0.49 | −0.49 | −0.44 | −0.44 | −0.49 | **−0.56^*^** | −0.35 | **−0.52^*^** | **−0.52^*^** | −0.31 | −0.38 | −0.45 | −0.40 | **−0.52^*^** |
| Fe | 0.32 | 0.26 | 0.25 | 0.36 | 0.30 | 0.26 | 0.27 | 0.35 | 0.28 | 0.26 | 0.29 | 0.30 | 0.27 | 0.38 |
| P | **−0.60^*^** | **−0.59^*^** | **−0.58^*^** | −0.42 | **−0.62^*^** | **−0.70^**^** | −0.39 | −0.42 | **−0.66^**^** | −0.40 | **−0.64^**^** | **−0.57^*^** | −0.18 | −0.46 |
| NH_4_^+^ | −0.16 | −0.10 | −0.14 | −0.12 | −0.20 | −0.20 | −0.06 | 0.02 | −0.21 | −0.26 | −0.25 | −0.16 | 0.13 | 0.03 |
| NO_3_^−^ | **−0.55^*^** | **−0.53^*^** | −0.50 | **−0.54^*^** | **−0.52^*^** | **−0.60^*^** | −0.41 | −0.34 | **−0.54^*^** | −0.36 | **−0.58^*^** | **−0.59^*^** | −0.41 | −0.48 |
| DOC | 0.36 | 0.43 | 0.45 | **0.51^*^** | 0.33 | 0.27 | 0.28 | **0.60^*^** | 0.34 | −0.11 | 0.33 | 0.35 | **0.53^*^** | 0.45 |
| CH_4_ | 0.04 | 0.03 | 0.01 | 0.01 | 0.00 | −0.03 | 0.22 | −0.10 | −0.13 | 0.21 | −0.04 | 0.00 | 0.36 | −0.13 |
| CO_2_ | 0.22 | 0.21 | 0.14 | −0.05 | 0.19 | 0.31 | 0.19 | −0.06 | 0.17 | 0.41 | 0.26 | 0.24 | 0.08 | 0.18 |
| N_2_O | **−0.58^*^** | **−0.58^*^** | **−0.60^*^** | **−0.60^*^** | **−0.65^**^** | **−0.67^**^** | −0.24 | **−0.58^*^** | **−0.73^**^** | 0.09 | **−0.57^*^** | **−0.67^**^** | −0.33 | **−0.57^*^** |

| **Table S3** Total nutrient content of the shoots and roots of maize plants grown in the soil from the different treatments after the soil recovery stage. Values represent mean ± standard error (n = 8 recipients per factor, 4 replications per combination of factors). Different letters indicate significant differences by using Tukey HSD post hoc test at 0.05 probability level. | | | | | | | | | | | | | |
| --- | --- | --- | --- | --- | --- | --- | --- | --- | --- | --- | --- | --- | --- |
|  |  | C | N | K | P | Ca | Mg | Al | Mn | Fe | Zn | Cu | Na |
|  |  | mg | mg | mg | mg | mg | mg | µg | µg | µg | µg | µg | mg |
|  |  | Shoot | | |  |  |  |  |  |  |  |  |  |
| Flood | C | 1123 ± 70 | 32.6 ± 2.7 | 46.1 ± 3.7 | 16.3 ± 1.4 | 9.0 ± 0.5 | 4.6 ± 0.3 | 272 ± 31 | 177 ± 13 | 182 ± 30 | 43 ± 4 | 20 ± 1 | 9 ± 1 |
|  | F | 1021 ± 74 | 33.4 ± 2.2 | 41.5 ± 2.7 | 14.9 ± 1.0 | 9.0 ± 0.8 | 4.4 ± 0.4 | 235 ± 28 | 169 ± 20 | 144 ± 25 | 41 ± 1 | 18 ± 3 | 9 ± 1 |
| *P* |  | 0.287 | 0.783 | 0.328 | 0.440 | 0.992 | 0.745 | 0.385 | 0.588 | 0.282 | 0.584 | 0.499 | 0.904 |
| Maize residue | NR | 1044 ± 91 | 37.0 ± 2.4 a | 42.7 ± 4.2 | 16.7 ± 1.3 | 8.6 ± 0.7 | 4.4 ± 0.4 | 255 ± 33 | 138 ± 13 b | 167 ± 33 | 45 ± 3 | 18 ± 2 | 8 ± 1 |
|  | MR | 1099 ± 50 | 29.0 ± 1.5 b | 44.9 ± 2.3 | 14.5 ± 1.0 | 9.3 ± 0.6 | 4.6 ± 0.3 | 252 ± 27 | 208 ± 6 a | 159 ± 23 | 40 ± 1 | 20 ± 2 | 9 ± 1 |
| *P* |  | 0.557 | **0.018** | 0.632 | 0.232 | 0.232 | 0.612 | 0.946 | **<0.001** | 0.827 | 0.124 | 0.456 | 0.473 |
|  | NR | 1197 ± 131 a |  |  |  |  |  |  |  | 229 ± 50 a |  | 22 ± 2 a |  |
|  | NR+F | 892 ± 78 a |  |  |  |  |  |  |  | 104 ± 5 a |  | 14 ± 2 a |  |
|  | MR | 1048 ± 43 a |  |  |  |  |  |  |  | 134 ± 12 a |  | 18 ± 1 a |  |
|  | MR+F | 1150 ± 90 a |  |  |  |  |  |  |  | 184 ± 43 a |  | 22 ± 4 a |  |
| Interaction (*P*) |  | **0.045** | 0.467 | 0.137 | 0.504 | 0.168 | 0.056 | 0.156 | 0.063 | **0.023** | 0.102 | **0.041** | 0.186 |
|  |  | Root |  |  |  |  |  |  |  |  |  |  |  |
| Flood | C | 578 ± 44 | 14.8 ± 1.0 | 23.6 ± 1.8 | 9.6 ± 1.3 | 8.0 ± 1.4 | 3.6 ± 0.4 | 4997 ± 825 | 372 ± 40 | 4909 ± 897 | 49 ± 6 | 29 ± 3 | 7 ± 1 |
|  | F | 561 ± 35 | 14.1 ± 0.7 | 22.3 ± 1.4 | 8.9 ± 1.0 | 5.9 ± 0.3 | 3.1 ± 0.3 | 4333 ± 518 | 272 ± 25 | 3763 ± 515 | 46 ± 3 | 25 ± 2 | 7 ± 1 |
| *P* |  | 0.749 | 0.396 | 0.481 | 0.445 | 0.168 | 0.177 | 0.289 | 0.059 | 0.123 | 0.560 | 0.283 | 0.566 |
| Maize residue | NR | 595 ± 48 | 16.2 ± 0.7 a | 25.7 ± 1.5 a | 11.9 ± 0.7 a | 8.3 ± 1.4 a | 4.0 ± 0.3 a | 6079 ± 550 a | 321 ± 49 | 5773 ± 692 a | 56 ± 5 a | 28 ± 3 | 7 ± 1 |
|  | MR | 543 ± 27 | 12.7 ± 0.5 b | 20.2 ± 1.0 b | 6.7 ± 0.5 b | 5.6 ± 0.3 b | 2.6 ± 0.1 b | 3251 ± 329 b | 323 ± 23 | 2900 ± 308 b | 39 ± 3 b | 26 ± 2 | 7 ± 1 |
| *P* |  | 0.343 | **0.001** | **0.012** | **<0.001** | **0.045** | **0.001** | **<0.001** | 0.978 | **0.001** | **0.005** | 0.367 | 0.686 |
| Interaction (*P*) |  | 0.092 | 0.165 | 0.365 | 0.609 | 0.102 | 0.185 | 0.116 | 0.226 | 0.174 | 0.053 | 0.089 | 0.133 |
| C: control without flood; F: Flood; NR: no-residue application; MR: Maize residue application (8 Mg ha^−1^). | | | | | | | | | | | | | |
